# Obstetric-Related Emergency Medical Treatment and Labor Act Violations and No Health Exception Bans

**DOI:** 10.1001/jamahealthforum.2025.4726

**Published:** 2025-12-05

**Authors:** Liana R. Woskie, Nora Brower, Jonathan Shaffer, Keren Ladin

**Affiliations:** 1 Department of Community Health, Tufts University, Medford, Massachusetts; 2 Department of Sociology, University of Vermont

## Abstract

**Importance:**

The Emergency Medical Treatment and Labor Act (EMTALA) guarantees access to emergency care, including for pregnancy-related conditions. Many states have enacted abortion bans that may conflict with the federal mandate under EMTALA to screen and stabilize patients in obstetric emergencies.

**Objective:**

To evaluate changes in obstetric-related EMTALA violations following state and federal restrictions on abortion.

**Design and Settings:**

This study used a staggered difference-in-differences design and Callaway-Sant’Anna estimator, leveraging a 100% sample of EMTALA filings from 2018 through quarter 1 of 2023 that was obtained via the Freedom of Information Act from the US Centers for Medicare & Medicaid Services. Filings were classified by clinical context and associated with stringency of state abortion laws. Additional models incorporated emergency department (ED) utilization data from the Healthcare Cost and Utilization Project and Medicaid expansion status to understand if violation trends were associated with shifting utilization patterns. Data were analyzed from February to July 2025.

**Exposures:**

State-level abortion bans that prohibit abortion and lack a clinically meaningful health exception for the pregnant person. Treatment was defined as adoption of a total or near-total abortion ban that (1) were effectively comprehensive across gestation, (2) provided no meaningful health exception, and (3) generated a documented allegation against preemption of federal EMTALA guidance during the study period.

**Main Outcomes and Measures:**

The primary outcome was the count of EMTALA violations involving obstetric emergencies. Secondary analyses examined ED utilization trends and the distribution of violations by infraction type (eg, failure to provide a medical screening examination).

**Results:**

States that enacted abortion bans with no health exception experienced a substantial increase in the number of EMTALA violations that were obstetric related, with an additional 1.18 violations per quarter (95% CI, 0.49-1.86; *P* = .001). Texas, which implemented Senate bill 8 in 2021, showed an average treatment effect on the treated of 0.69 violations per quarter (95% CI, 0.46-0.91; *P* < .001). The 5 states that triggered no health exception bans had a smaller, imprecise increase (0.49; 95% CI, –0.13 to 1.12; *P* = .12). A concurrent rise in ED utilization in non-Medicaid expansion states and a growing number of obstetric violations due to failures in medical screening were also observed, suggesting delays in initial triage or assessment.

**Conclusions and Relevance:**

The results of this difference-in-differences analysis suggest that strict abortion bans, particularly Senate bill 8 in Texas, were followed by a measurable escalation in EMTALA violations involving obstetric emergencies. Uncertainty regarding state policies and deferral to state law may reshape frontline emergency care and compromise federally protected access in obstetric emergencies.

## Key Points

**Question:**

How did obstetric-related Emergency Medical Treatment and Labor Act (EMTALA) violations change in association with state-level abortion policy?

**Findings:**

In this difference-in-differences analysis of EMTALA violations from 2018 to the first quarter of 2023, states with no health exceptions saw a substantial rise in obstetric-related violations that were associated with policies adopted by Texas. There was a concurrent rise in emergency department utilization, and screening failures suggest that violations may have increasingly occurred on arrival before treatment.

**Meaning:**

The study results suggest that state abortion laws may undermine federally protected care in obstetric emergencies.

## Teaser

This difference-in-differences analysis examines changes in obstetric-related Emergency Medical Treatment and Labor Act violation filings following state and federal restrictions on abortion.

## Introduction

The Emergency Medical Treatment and Labor Act (EMTALA) is a federal statute that requires all Medicare-participating hospitals with emergency departments (EDs) to screen and stabilize patients who present with an emergency medical condition.^1,2^ EMTALA applies to all emergency conditions, including pregnancy-related complications.^3^ In 2022, the US Supreme Court’s decision in *Dobbs v Jackson Women’s Health Organization* (*Dobbs*) eliminated federal constitutional protections for abortion and returned authority regarding abortion policy to individual states.^4^ Several states enacted laws that restricted abortion access following the *Dobbs* decision, while some, including Texas, enacted abortion restrictions such as Senate bill 8 (SB8), which preceded the overturn of *Roe v Wade*.^5^ These regulations present conflicts for clinicians faced with adhering to the federal mandate of EMTALA^6,7^ and have generated substantial legal debate and clinical uncertainty as to how to reconcile federal care requirements with restrictive state-level abortion bans.^8,9,10^

Ambiguity regarding federal EMTALA obligations vs state guidance for abortion has implications for emergency and broader obstetric practice.^11^ For example, the timely administration of misoprostol in the context of miscarriage management may be necessary to prevent sepsis or other serious morbidities, underscoring the relevance of EMTALA in emergency obstetric contexts.^12^ Although a rich legal scholarship has addressed the intersection of EMTALA and state-level abortion restrictions,^13,14,15^ including in post-*Dobbs* litigation (eg, *United States v Idaho*), empirical research on how EMTALA has actually functioned in this policy environment remains limited.^10^ A recent analysis suggested that up to 12% of EMTALA violations before COVID-19 involved obstetric or gynecologic care.^16^ Qualitative studies indicate that clinicians in states with restrictive abortion laws experienced increased uncertainty about their obligations under EMTALA, potentially leading to delays or denials of care.^17^ This uncertainty was further compounded by variation in statutory language: while some states permit abortion if the pregnant person’s life is at risk, fewer provide exceptions for threats to health. Even when health exceptions do exist, legal framing, such as references to “permanent impairment of a life-sustaining organ,” may lack clinical clarity, making real-time decision-making difficult.^8^ In this context, clinicians have expressed fear of legal repercussion for providing medically indicated care, which could itself constitute an EMTALA violation.^3^ Despite the increase in these conflicting obligations and ambiguity of guidance for clinicians, little is known about how EMTALA violations have changed over time or across states with no health exception laws.

To address this gap, we analyzed trends in obstetric-related EMTALA violations before and after restrictive state policies, focusing on states with abortion bans that lack a meaningful health-related exception (beyond a threat to life) for the pregnant person. This informed the following research questions: first, to what degree did trends in EMTALA violations differ following restrictive state-level policies, accounting for ED patterns and Medicaid expansion? Second, did obstetric-related EMTALA violations change by infraction type (eg, stabilization)?

## Methods

### Data Sources and Variables

The Tufts Health Sciences institutional review board reviewed this project and determined that it did not constitute research involving human participants as defined by federal regulations; thus, formal institutional review board approval and participant-informed consent were waived. The study was reported according to the Strengthening the Reporting of Observational Studies in Epidemiology (STROBE) reporting guideline. We used data on all EMTALA investigation records from quarter 1 of 2018 through quarter 1 of 2023, as obtained via a Freedom of Information Act request to the US Centers for Medicare & Medicaid Services (CMS). Data were analyzed from February to July 2025. CMS, which screens and investigates EMTALA complaints, provided a dataset containing violation filing records, which was released without redaction. Each observation corresponds to an EMTALA investigation and includes the hospital’s CMS certification number, state, federal region, and survey exit date. Variables include investigation outcomes (violation vs no violation), infraction tag (eg, A2400 policies and procedures, A2402 signage, A2405 central log, A2406 medical screening examination, A2407 stabilizing treatment, A2409 appropriate transfer, and A2411 acceptance of transfers), and emergency type (eg, medical, psychiatric, labor related, other obstetric, and surgical). Violations are primarily reported through a complaint-driven process initiated by individuals, including patients, health care workers, or even other hospitals, to the CMS or the relevant state survey agency. Hospitals are required to report suspected EMTALA violations of other hospitals within 72 hours. When investigators identify noncompliance, CMS issues an EMTALA violation (hereafter violation) and assigns 1 or more deficiency tags. We classified violations whose tags referenced labor or other obstetric diagnoses as obstetric-related EMTALA violations and used this as our primary outcome. This outcome was defined at the state policy level and likely operationalized through hospital directives, not individual (ie, physician) statutory knowledge.

We augmented these data with state-level abortion policy information through a 4-step abstraction process. First, we constructed a universe of near-total abortion prohibitions by querying Westlaw Edge Statutes and Regulations; bill texts were downloaded from state legislative sites and effective date clauses were cross-checked against 3 reproductive-specific trackers (Guttmacher Institute, Kaiser Family Foundation, and the Center for Reproductive Rights). Second, each statute was linked to an alleged conflict with EMTALA by searching (1) the CMS Survey & Certification portal, (2) Public Access to Court Electronic Records and Bloomberg Law dockets and US Department of Justice releases for federal pre-emption actions, (3) state attorney general advisories citing EMTALA, and (4) LexisNexis and hospital association newsletters for institutional compliance bulletins. Third, to ensure adequate observation time, we tracked when laws were enjoined or otherwise inoperative using the Bloomberg Law track legislation feature that was validated against the reproductive-specific policy trackers. Together, state-specific statutory text, alleged conflict, and enforcement dates (including when each ban was enacted) were abstracted into a master file.

We also used the Healthcare Cost and Utilization Project (HCUP) Fast Stats tool, which provides aggregated data derived from the Nationwide Emergency Department Sample, State Emergency Department Databases, and State Inpatient Databases. These data include treat-and-release visits and those resulting in admission. Counts are stratified by payer (all payer, Medicaid and self-pay, self-pay only); HCUP data are regularly used to assess shifting ED patterns.^18,19,20^

### Policy Treatment

We defined treatment as adoption of a total or near-total abortion ban that (1) was effectively comprehensive across gestation, (2) provided no meaningful health exception, and (3) generated a documented allegation against preemption of federal EMTALA guidance during the study period. Six states (Idaho, Kentucky, Louisiana, Mississippi, Oklahoma, and Texas) met these criteria (Table 1). Regarding meaningful health exemptions, SB8 from Texas illustrates the issue. Although the statute permits abortion to avert “serious risk of death or major bodily-function impairment,” CMS issued deficiency letters to 2 Texas hospitals after patients with ectopic pregnancy or previable membrane rupture were denied care.^21^ In turn, clinicians and legal scholars have described unduly narrow medical emergency carve-outs as functionally unusable.^8,9,22^ In this analysis, these bans are considered, and referred to, as *no health exception*.

**Table 1.** Classification of US States by Statutory Profile Relevant to Abortion and EMTALA From 2018 to 2023 Q1

Group	States	Statutory profile	Use in study
Treated group: total or near-total abortion bans with no meaningful health exception, an EMTALA conflict, and adequate data	Idaho, Kentucky, Louisiana, Mississippi, Oklahoma, and Texas (n = 6)^a^	Total or near-total bans that (1) apply across gestations; (2) limit care to preventing death or averting a substantial and irreversible impairment of a major bodily function, with mental health expressly excluded; and (3) an alleged conflict between state and federal law (eg, DOJ preemption actions challenging statutory language in state abortion laws or state attorney-general guidance)	Treated = 1; from each statute’s activation date (eg, Texas 2021, Q3) in primary specifications
Potentially treated group: similar bans, but no documented EMTALA conflict/inadequate data	Arkansas, South Dakota, Alabama, North Dakota, Missouri, Tennessee, and West Virginia (n = 7)^b^	State statutes meet items 1 and 2 but have no documented EMTALA preemption/enforcement action from 2018 to 2023 or supplied 3 or fewer consecutive postban quarters with any obstetric EMTALA investigations	Excluded: excluded from the control pool; included only in sensitivity analyses
Controls: all other states with data	34 States and Washington DC (n = 35)^c^^,^^d^	States that have no post-Dobbs total ban or bans/gestational limits that retain an explicit physical (and, in many cases, mental) health exception consistent with EMTALA	Treated = 0; control group in primary analyses

Abbreviations: DOJ, US Department of Justice; EMTALA, Emergency Medical Treatment and Labor Act; Q, quarter.

^a^ Treatment cohort (n = 6). States enforced a near-total abortion prohibition that (1) are effectively comprehensive across gestation; (2) limited care to preventing maternal death or averting a substantial and irreversible impairment of a major bodily function, with mental health indications excluded; and (3) an alleged conflict between state and federal law (eg, DOJ pre-emption actions challenging statutory language in state abortion laws or state attorney-general guidance). Each statute remained operative for 3 or more consecutive quarters to ensure adequate postpolicy observation.

^b^ Potentially treated states (n = 7). Arkansas, South Dakota, Alabama, North Dakota, Missouri, Tennessee, and West Virginia enacted narrow or ambiguous physical health exceptions but either failed to trigger an EMTALA enforcement action from 2018 to 2023 or provided fewer than 3 consecutive postban quarters with any obstetric-related EMTALA investigation. These states are excluded from the primary control pool and appear only in sensitivity analyses.

^c^ Control cohort (n = 35). All remaining jurisdictions with qualifying hospital-year observations retained either no post-Dobbs abortion ban or gestational limits that include an explicit physical (and, in many cases, mental) health exception consistent with EMTALA. Control states: Arizona, California, Colorado, Connecticut, Delaware, Florida, Georgia, Illinois, Indiana, Iowa, Kansas, Maine, Maryland, Massachusetts, Michigan, Minnesota, Montana, Nebraska, Nevada, New Hampshire, New Jersey, New Mexico, New York, North Carolina, Ohio, Oregon, Pennsylvania, South Carolina, Utah, Vermont, Virginia, Washington, Wisconsin, Wyoming, and Washington DC.

^d^ Data exclusions. Alaska, Hawaii, and Rhode Island were dropped from all models because no eligible hospital-year observations were recorded during 2018 to 2023 Q1.

Activation dates were aligned with quarters (eg, Texas, quarter 3 of 2021). A state was treated only if the ban was operative for 3 or more consecutive quarters within the study period. If a ban was temporarily enjoined (eg, Louisiana, Kentucky, and Idaho), we followed a predominant exposure rule: the quarter was coded as treated if the ban was enforceable 50% or more days. Seven additional states enacted similarly narrow statutory language but either lacked a state-specific alleged conflict with EMTALA during the study period (Arkansas, South Dakota) or had insufficient postban observations (Alabama, North Dakota, Missouri, Tennessee, and West Virginia); these were excluded from the control pool. The remaining 34 states plus Washington, DC, retained broad physical or mental health exceptions throughout the period and formed the control group. Alaska, Hawaii, and Rhode Island contributed no qualifying hospital-year observations and were excluded.

### Empirical Approach

We used a staggered ordinary least-squares difference-in-differences (DiD) specification and a Callaway–Sant’Anna estimator^23^ to assess whether violations involving obstetric-related emergencies changed following restrictive state policies that lacked a health exception for pregnant people. We estimated the association between state-level no health exception bans and obstetric-related EMTALA deficiencies, as well as negative control outcomes and psychiatric, surgical, and medical deficiencies. All outcomes were summed at the state-quarter level, smoothing month-to-month variation.^23,24^ Model 1 was unadjusted and included the DiD interaction only; model 2 added state and calendar-quarter fixed effects; and model 3 further adjusted for all-payer ED visit volume. The Callaway-Sant’Anna staggered adoption estimator^23^ allowed us to augment primary DiD analyses for the obstetric outcome by generating group-specific average treatment effects on the treated (ATT) for Texas and the 5 *Dobbs*-activated states; a state-weighted pooled ATT was calculated. For all models, standard errors were heteroskedasticity robust and clustered at the state level to account for within-state correlation over time.^25^

To understand underlying shifts in ED utilization and account for nonparallel pretrends detected in the ED utilization models, we estimated postban changes using synthetic difference-in-differences (SDiD) weighting, a hybrid of the synthetic control and 2-way, fixed-effects designs that is valid with staggered policy adoption and multiple treated units.^26^ SDiD constructs unit-specific (state) and time-specific weights (quarter) that align the treated and control states on preintervention levels and trajectories and then applies those weights in a DiD estimator with state and calendar-quarter fixed effects.^23^ We ran models separately by Medicaid expansion status to examine heterogeneous effects. SDiD models were fit with cluster-robust standard errors at the state level. Therefore, point estimates represent the change in mean quarterly ED encounters per treated state compared with a synthetic control that reproduces prepolicy trends. As in the primary analyses, we retained state and quarter fixed effects but did not adjust for ED visits to avoid conditioning on the outcome. Infraction-type models (eg, medical screening examination violations) were reestimated under the fully adjusted specification used in model 3 because parallel pretrends were met for these outcomes (eTable 10 in Supplement 1). Analyses were conducted using Stata/MP, versions 18.0 and 19.5 (StataCorp), and Python, version 3.11 (Python Software Foundation). Two-sided *P* < .05 was considered statistically significant.

## Results

During the preintervention period, treatment states had a relatively similar annual number of EMTALA violations as controls (mean, 11.97 vs 16.49 per state; standardized mean difference [SMD], −0.29; Table 2). The clinical distribution of violation types was broadly comparable: medical emergencies constituted roughly two-thirds of deficiencies in both groups, and obstetric related accounted for less than 10%. Imbalances emerged for psychiatric events (25.0% vs 18.0%; SMD, 0.40) and surgical emergencies (0.0% vs 4.0%; SMD, −0.65). Deficiency tag profiles were similar, although treatment states had higher shares of appropriate transfer (A2409) and stabilizing treatment (A2407) citations. Average ED volume did not differ meaningfully during the preperiod, but treatment states had a smaller Medicaid plus self-pay share (31.0% vs 35.0%; SMD, −0.55), indicating a modestly different payer mix before policy implementation.

**Table 2.** Characteristics of Treatment and Control States During the Preintervention Period

Characteristic	States, mean (SD) %		Standardized mean difference
	Treatment: no health exception abortion bans (n = 6)	Control: never treated, excluding potentially treated (n = 35)^a^	
EMTALA violations			
Mean (SD) annual count per state	11.97 (19.79)	16.49 (14.58)	–0.29
Clinical type, violations per state^b^			
Medical	63.0 (12.0%)	67.0 (20.0)	–0.19
Psychiatric	25.0 (22.0%)	18.0 (16.0)	0.40
Obstetric-related	7.0 (8.0%)	9.0 (8.0)	–0.31
Surgical	0	4.0 (6.0)	–0.65
Other	2. (5.0)	6.0 (11.0)	–0.34
Deficiency tags, violations per state			
Medical screening examination (tag A2406)	28.6 (4.0)	27.0 (7.9)	0.22
General compliance (tag A2400)	26.5 (18.3)	26.1 (14.0)	0.03
Appropriate transfer (tag A2409)	16.0 (16.1)	11.8 (8.0)	0.44
Stabilizing treatment (tag A2407)	13.9 (7.1)	9.2 (7.9)	0.60
ED log maintenance (tag A2405)	8.5 (10.0)	10.3 (6.5)	–0.26
ED visits			
All payers, mean (SD) visits per state	696 881 (953 446)	606 632 (634 892)	0.13
Payer mix, visits per state^b^			
Medicaid + self-pay	31.0 (10.0)	35.0 (6.0)	–0.55

Abbreviations: ED, emergency department; EMTALA, Emergency Medical Treatment and Labor Act.

^a^ Excludes 7 states with ambiguous no health exception abortion bans that are considered potentially treated (Alabama, Missouri, North Dakota, Tennessee, and West Virginia, plus 2 primary-treated states already counted in the treatment group).

^b^ State-year totals for Hospital Cost and Utilization Project data. Shares calculated within state-year then averaged across years.

### No Health Exception Policies and EMTALA Violations

In staggered DiD models, treated states experienced an overall rise in obstetric EMTALA violations after adopting abortion bans without a health exception (Figure). The mean excess was 0.50 violations per state-quarter in the unadjusted model (95% CI, 0.09-0.90; *P* = .02), 0.70 after adding state and calendar-quarter fixed effects (95% CI, 0.19-1.20; *P* = .01), and 1.18 with additional adjustment for ED visit volume (95% CI, 0.49-1.86; *P* = .001). Corresponding changes in medical (0.35; 95% CI, –2.00 to 2.69; *P* = .77) and surgical (0.21; 95% CI, –0.11 to 0.53; *P* = .20) violations were small and nonsignificant, indicating the policy effect was confined to obstetric emergencies.

**Figure.**
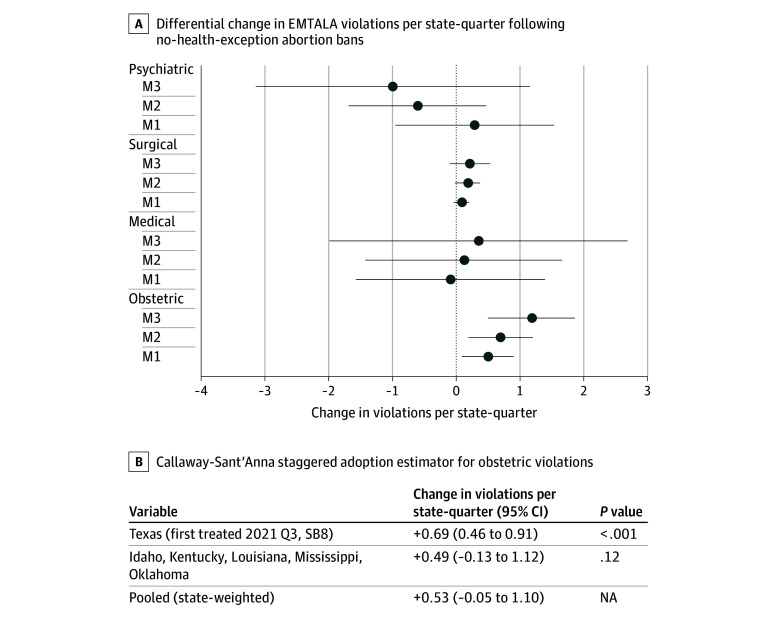
Primary Staggered Difference-in-Differences Estimates for Emergency Medical Treatment and Labor Act (EMTALA) Violations From 2018 to the First Quarter of 2023 A, Differential change in EMTALA violations per state-quarter following no health exception abortion bans. Points represent the estimated change (Δ) in violations per state-quarter for each patient condition category as derived from 3 difference-in-differences model specifications; error bars denote 95% CIs. Model (M) 1 is unadjusted; model 2 adds state and calendar-quarter fixed effects; and model 3 additionally adjusts for all-payer emergency department visit volume (Hospital Cost and Utilization Project data). Positive values indicate an increase in violations in association with the counterfactual trend. B, Callaway-Sant’Anna staggered adoption estimator for obstetric violations. Group-specific average treatment effects on the treated estimated with state and quarter fixed effects; never-treated states serve as controls, and already treated states are removed from the control set after their own adoption. The pooled effect is weighted by the number of treated states in each group. NA indicates not applicable; SB8, Senate bill 8; Q, quarter.

The Callaway–Sant’Anna estimator confirmed underlying heterogeneity. Texas, first treated in quarter 3 of 2021 (SB8), showed an ATT of 0.69 violations per quarter (95% CI, 0.46-0.91; *P* < .001). The 5 states that triggered bans after *Dobbs* had a smaller and imprecise excess (0.49; 95% CI, −0.13 to 1.12; *P* = .12). When the 2 groups were combined, the state-weighted pooled ATT was 0.53 violations (95% CI, −0.05 to 1.10). Thus, the aggregate increase was largely associated with Texas’s early adoption, whereas later-adopting states displayed a directionally similar but statistically nonsignificant pattern.

### Shifting ED Utilization and Medicaid Expansion

Using staggered synthetic DiD analyses, we found that no health exception policies were followed by a change in ED encounters (Table 3). Medicaid expansion states (Idaho, Kentucky, Louisiana, and Oklahoma) saw a nonsignificant mean quarterly change of 1701 Medicaid and self-pay visits (95% CI, –11 769 to 15 171; *P* = .80) and –15 739 all-payer visits (95% CI, –44 666 to 13 188; *P* = .29). In states that did not expand Medicaid (Mississippi and Texas), Medicaid and self-pay ED encounters increased by 11 302 per state-quarter (95% CI, 3507 to 19 097; *P* = .01) and all-payer encounters rose by 33 610 (95% CI, 7218 to 60 002; *P* = .01) after no health exception abortion bans took effect. Point estimates reflect the mean change for a treated state compared with its synthetic control. The rise in ED encounters among nonexpansion states was primarily associated with Texas, which adopted its ban earlier and has a substantially higher baseline ED volume. Mississippi showed a concordant but smaller increase; its inclusion narrowed the confidence interval without altering the overall direction of effect.

**Table 3.** Differential Change in Emergency Department (ED) Utilization in No Health Exception States by Payer Type, With and Without Medicaid Expansion^a^

Visits	Treatment states			
	Expansion: Idaho, Kentucky, Louisiana, and Oklahoma^b^		Nonexpansion: Mississippi and Texas	
	State-quarter mean change (95% CI)	*P* value	State-quarter mean change (95% CI)	*P* value
Medicaid and self-pay ED visits	+1701 (−11 769 to 15 171)	.80	11 302 (3507 to 19 097)	.01
All-payer ED visits	–15 739 (−44 666 to 13 188)	.29	33 610 (7218 to 60 002)	.01

^a^ Synthetic difference-in-differences estimates construct unit-specific and period-specific weights that align the treated and control states on preintervention levels and trajectories then apply those weights in a difference-in-differences estimator with state and calendar-quarter fixed effects. β Coefficients depict the change in mean quarterly ED encounters per treated state after restrictive state policies vs weighted never-treated controls. 95% CIs were derived from state-clustered standard errors. Models include state and calendar-quarter fixed effects and divide the 6 treated states into Medicaid expansion (Idaho, Kentucky, Louisiana, and Oklahoma) and nonexpansion (Mississippi and Texas) groups, using staggered policy dates for the post indicator.

^b^ Expansion: Idaho (2020), Kentucky (2014), Louisiana (2016), and Oklahoma (2021).

### Infraction Types for Obstetric Violations

Compared with controls, treatment states recorded a significant postban rise in medical screening examination citations (0.42 violations per state-quarter; 95% CI, 0.19-0.65; *P* < .001) and general compliance citations (0.25; 95% CI, 0.04-0.46; *P* = .02; Table 4). Changes for appropriate transfer, stabilizing treatment, and ED log maintenance (A2405) counts were not statistically significant. Thus, the overall growth in obstetric EMTALA violations appeared to be principally associated with deficiencies in initial medical screening and basic compliance with §489.24. However, null findings cannot rule out policy-relevant increases of 0.1 to 0.2 violations per quarter, as the study was underpowered to detect changes of this size with confidence.

**Table 4.** Differential Change in EMTALA Violation Types Following No Health Exception Abortion Bans^a^

EMTALA deficiency tag	Δ Violations per state-quarter (95% CI)	*P* value
Medical screening examination (2406)	0.42 (0.19 to 0.65)	<.001
General compliance (2400)	0.25 (0.04 to 0.46)	.02
Appropriate transfer (2409)	0.08 (–0.02 to 0.19)	.10
Stabilizing treatment (2407)	0.09 (–0.14 to 0.33)	.43
ED log maintenance (2405)	0.08 (–0.20 to 0.36)	.57

Abbreviations: ED, emergency department; EMTALA, Emergency Medical Treatment and Labor Act.

^a^ Values are average treatment effects on the treated from staggered difference-in-differences models, expressed as the absolute change (Δ) in quarterly counts of tag-specific obstetric EMTALA violations per state. Six treatment states were compared with 35 states without abortion bans that served as controls (extended-ban states excluded). All models included state and calendar-quarter fixed effects and were adjusted for mean all-payer ED visits; standard errors were clustered by state. A 2-sided *P* < .05 was considered statistically significant.

## Discussion

Obstetric-related violations increased after states enacted abortion bans that lacked a meaningful health exception, rising by 1.18 obstetric violations per state-quarter (95% CI, 0.49-1.86) compared with controls. The increase was largely associated with Texas, where SB8 preceded *Dobbs*; the 5 post-*Dobb*s states showed a smaller, not statistically significant increase in isolation. These patterns were unique to obstetric violations: similar patterns were not observed for nonobstetric EMTALA violations. This supports the hypothesis that state abortion policies, rather than secular reporting trends, informed the increase. We also observed corresponding increases in overall ED utilization in no health exception ban states that had not expanded Medicaid and a growing failure to provide medical screenings to obstetric patients in the ED.

While preliminary, these data point to early breakdowns in care (ie, at the point of entry, when evaluation and triage should occur). Failure to screen violations may indicate that patients were not assessed on arrival, which is consistent with reports of individuals turned away or told to return only if their condition worsened.^27^ This pattern may reflect fundamental issues of clinical hesitancy or institutional protocols that inhibit physicians or other frontline staff from giving even the most basic care in obstetric emergencies.^28^ Limited familiarity and confusion given evolving state statutes may further delay assessment, consultation, or transfer for pregnancy-related emergencies, thereby contributing to the violations we have observed.

Texas accounted for more than half of the aggregate excess in obstetric EMTALA violations (0.69 per state-quarter). Two intersecting factors may have amplified the effect. First, SB8 produced the longest exposure window and uniquely empowered private enforcement, heightening legal uncertainty months before *Dobbs*.^29,30^ Second, neither Texas nor Mississippi had adopted Medicaid expansion. Although expansion does not alter pregnancy-specific Medicaid eligibility, prior research has suggested that it likely narrows the coverage gap for low-income individuals before conception and after the 60-day postpartum cutoff, reduces uninsured ED use, and may improve hospital margins that fund obstetric call coverage and compliance training relevant to EMTALA.^31,32^ Prior studies have identified Texas as a harbinger of future outcomes in states that enacted strict abortion laws following *Dobbs*. Gemmill et al^33^ found that a 6-week abortion ban in Texas was associated with a 13% increase in the number of infants who died during their first year of life. Because Texas enacted its abortion restrictions in September 2021 before the overturning of *Roe v Wade*, scholars and policymakers have used Texas as a case study for estimating outcomes for the rest of the US. Early evidence following *Dobbs* suggests the rise in infant mortality observed in Texas is predictive of outcomes in the US.^34^ Drawing on these findings, our study presents a framework for future research to examine implications of EMTALA violations and ED utilization in the coming years.

Rising violations persisted after adjusting for ED use, but the shifting ED landscape is important to examine in association with abortion restrictions. Treatment states had higher uninsured and self-pay ED visit rates before no health exception policies and likely experienced additional strain from reproductive health clinic closures.^35^ While not yet fully understood, these closures may reorient baseline care to the ED or delay care seeking, leading to higher-acuity presentation.^33,36^ As frontline reproductive care is restricted, the role of the ED and EMTALA as a safeguard is increasingly relevant. Recent litigation, such as *United States v Idaho* and *St Luke’s v Labrador*, has underscored how state-level abortion bans may conflict with long-standing emergency care obligations.^37,38^ Our results suggest that despite federal preemption, hospitals may already be deferring to state laws when the 2 conflict, highlighting the need for accountability mechanisms for federal rights violations in the post-*Dobbs* era.^8^

### Limitations

This article had several limitations. First, obstetric-related EMTALA violations remain exceedingly rare events, which raises concerns of sensitivity to small shifts and prohibited us from examining single states apart from Texas. Results from single-coefficient DiDs should be interpreted as blunt and, given the short postpolicy period, preliminary. They may change if high-population states, such as Florida, enact similar bans. In addition, we do not know if obstetric violations were specific to abortion; however, there is reason to believe that post-*Dobbs* care disruptions extended beyond abortion to affect the broader management of pregnancy-related emergencies.^17^ Third, our secondary outcome, ED utilization, has partial missingness, a short postperiod, and lacks visit-level granularity, preventing isolation of pregnancy-specific visits. Fourth, EMTALA relies on patient and physician reporting, which may rise with advocacy support following *Dobb*s. Finally, several treatment states fall within the same CMS administrative region, and while we included state fixed effects, geographic clustering may have raised residual confounding.

## Conclusions

Although the US does not grant a constitutional right to health care, EMTALA provides a rare statutory guarantee for emergency medical access. It obligates hospitals to evaluate and stabilize patients in emergency contexts. In this study with a DiD analysis, states with no health exception abortion laws, especially Texas, experienced an increase in obstetric-related EMTALA violations, including failure to provide medical screenings. Our findings suggest that legal and institutional uncertainty may contribute to new breakdowns and delays in emergency obstetric care, threatening the foundational promise of EMTALA, which is the right to be treated in an emergency.

## Notes

Supplement 1. eTable 1. Emergency Medical Treatment and Labor Act (EMTALA) Deficiency Tags Identified in the Study Sample and Their Regulatory Interpretations eTable 2. Descriptive profile of EMTALA citations in the analytic file eTable 3. Patterns across states eTable 4. Data Underlying Table 1 eMethods 1. Emergency Department (ED) Data Completeness and Missingness eMethods 2. Rationale for Including State Medicaid Expansion Status in Models of ED Utilization Related to Obstetric-Related EMTALA Violations eTable 5. Medicaid Expansion Status by State eTable 6. Potential Mechanisms Underlying post-Policy ED Trends in Texas and Mississippi eMethods 3. Identification of State Abortion Policies that Conflict with EMTALA eMethods 4. Overview of Sources & Processes to Characterize State Policy eTable 7. Application of Inclusion/Exclusion Rules, 2018 Q1-2023 Q1 eTable 8. “Documented EMTALA conflict” as inclusion criteria eTable 9. Joint Parallel-Trend Test for the Primary Obstetric-Related Difference-in-Differences Model eTable 10. Tag-Specific “Violation Type” Difference-in-Differences Models eTable 11. Point estimates underlying Figure 1, primary DiD models

Supplement 2. Data sharing statement
