# Supplementary material for: Obstetric-Related Emergency Medical Treatment and Labor Act Violations and No Health Exception Bans
Source: JAMA Health Forum. 2025 Dec 5;6(12):e254726. doi: 10.1001/jamahealthforum.2025.4726 (PMC12681040; doi:10.1001/jamahealthforum.2025.4726)
Supplement: Supplement 1. — eTable 1. Emergency Medical Treatment and Labor Act (EMTALA) Deficiency Tags Identified in the Study Sample and Their Regulatory Interpretations eTable 2. Descriptive profile of EMTALA citations in the analytic file eTable 3. Patterns across states eTable 4. Data Underlying Table 1 eMethods 1. Emergency Department (ED) Data Completeness and Missingness eMethods 2. Rationale for Including State Medicaid Expansion Status in Models of ED Utilization Related to Obstetric-Related EMTALA Violations eTable 5. Medicaid Expansion Status by State eTable 6. Potential Mechanisms Underlying post-Policy ED Trends in Texas and Mississippi eMethods 3. Identification of State Abortion Policies that Conflict with EMTALA eMethods 4. Overview of Sources & Processes to Characterize State Policy eTable 7. Application of Inclusion/Exclusion Rules, 2018 Q1-2023 Q1 eTable 8. “Documented EMTALA conflict” as inclusion criteria eTable 9. Joint Parallel-Trend Test for the Primary Obstetric-Related Difference-in-Differences Model eTable 10. Tag-Specific “Violation Type” Difference-in-Differences Models eTable 11. Point estimates underlying Figure 1, primary DiD models [file jamahealthforum-e254726-s001.pdf]

Supplemental Online Content

Woskie LR, Brower N, Shaffer J, Ladin K. Obstetric-related Emergency Medical Treatment and Labor Act violations and no health exception bans. *JAMA Health Forum*. 2025;6(12):e254726. doi:10.1001/jamahealthforum.2025.4726

eTable 1. Emergency Medical Treatment and Labor Act (EMTALA) Deficiency Tags Identified in the Study Sample and Their Regulatory Interpretations
eTable 2. Descriptive profile of EMTALA citations in the analytic file
eTable 3. Patterns across states
eTable 4. Data Underlying Table 1
eMethods 1. Emergency Department (ED) Data Completeness and Missingness
eMethods 2. Rationale for Including State Medicaid Expansion Status in Models of ED Utilization Related to Obstetric-Related EMTALA Violations
eTable 5. Medicaid Expansion Status by State
eTable 6. Potential Mechanisms Underlying post-Policy ED Trends in Texas and Mississippi
eMethods 3. Identification of State Abortion Policies that Conflict with EMTALA
eMethods 4. Overview of Sources & Processes to Characterize State Policy
eTable 7. Application of Inclusion/Exclusion Rules, 2018 Q1-2023 Q1
eTable 8. “Documented EMTALA conflict” as inclusion criteria
eTable 9. Joint Parallel-Trend Test for the Primary Obstetric-Related Difference-in-Differences Model
eTable 10. Tag-Specific “Violation Type” Difference-in-Differences Models
eTable 11. Point estimates underlying Figure 1, primary DiD models

This supplemental material has been provided by the authors to give readers additional information about their work.

© 2024 Woskie LR et al. JAMA Health Forum.

eTable 1. Emergency Medical Treatment and Labor Act (EMTALA) Deficiency Tags Identified in the Study Sample and Their Regulatory Interpretations

Infraction Type	Description	Interpretation
**A2400 – General Policies and Procedures**	"General" i.e. Hospital lacks or fails to implement policies ensuring compliance with EMTALA requirements.	Reflects systemic issues in institutional compliance, potentially leading to multiple downstream violations.
**A2402 – Signage**	Failure to post required signage informing patients of their rights under EMTALA.	May deter patients from seeking emergency care, violating patient notification obligations.
**A2405 – Central Log**	Failure to maintain a central log of individuals who come to the emergency department seeking treatment.	Indicates administrative lapses in tracking patient encounters, hindering accountability and compliance monitoring.
**A2406 – Medical Screening Examination (MSE)**	Failure to provide an appropriate medical screening examination to determine if an emergency medical condition exists.	Represents a core EMTALA violation, potentially leading to missed diagnoses and inadequate care.
**A2407 – Stabilizing Treatment**	Failure to provide necessary stabilizing treatment for patients with emergency medical conditions.	Can result in patient harm due to deterioration of condition, and is a serious breach of EMTALA obligations.
**A2409 – Appropriate Transfer**	Failure to arrange an appropriate transfer for patients with emergency medical conditions.	Includes inadequate documentation, lack of consent, or transferring unstable patients without meeting regulatory criteria.
**A2411 – Acceptance of Transfers**	Failure of a hospital with specialized capabilities to accept an appropriate transfer of a patient requiring such services.	Violates the obligation to accept transfers when the hospital has the capacity and capability to treat the patient.

Tag numbers (A2400–A2411) correspond to the Centers for Medicare & Medicaid Services (CMS) State Operations Manual, Appendix V, which enumerates surveyor guidelines for enforcing EMTALA (42 CFR §489.24). "Likely interpretation" summarizes how CMS surveyors classify the cited deficiency on inspection reports. Abbreviations: CMS, Centers for Medicare & Medicaid Services; EMTALA, Emergency Medical Treatment and Labor Act; MSE, medical screening examination.

© 2024 Woskie LR et al. JAMA Health Forum.

Rare Outcome

eTable 2. Descriptive profile of EMTALA citations in the analytic file

**Metric**	**All EMTALA violations**	**Obstetric-related violations***
Total number of citation records	3,747	430
State-quarters in study window	1,344 (48 jurisdictions × 28 quarters)	same
State-quarters with ≥ 1 citation	676 (50.1 %)	127 (9.5 %)
Range of citations per state-quarter	0 – 31	0 – 13
Median (IQR) citations per state-quarter†	1 (0 – 4)	0 (0 – 0)
Mean ± SD citations per state-quarter	2.8 ± 4.1	0.29 ± 1.0

**Defined by the obs_violation flag (includes labour- and other obstetric-emergency tags).*
†Includes zero-citation quarters; among state-quarters with ≥1 citation the medians are 4 (IQR 2–8) for all EMTALA and 2 (IQR 1–3) for obstetric citations.

eTable 3. Patterns across states

State (top 10 by total citations)	**Cumulative EMTALA citations**	**Median citations per state-quarter**	**Maximum in any quarter**
Tennessee	267	9.5	28
North Carolina	265	8.0	31
Wisconsin	196	6.0	23
Missouri	194	7.0	21
California	182	6.5	18
Texas	**181**	**7.0**	**16**
Georgia	179	5.0	20
Florida	173	6.5	18
Washington	160	4.5	26
Oregon	140	4.0	21

Texas ranks 6-th overall but is one of only four states with a median ≥7 citations per quarter, confirming that populous jurisdictions contribute a disproportionate share of observations.

© 2024 Woskie LR et al. JAMA Health Forum.

Addressing Data Missingness

eTable 4. Data Underlying Table 1

Data element	Practical handling in the code	Rationale
Violation count (annual totals)	We counted rows with determination == "EMTALA Violation" for each state-year. If a state truly has zero violations in a given calendar year, that zero is kept.	The EMTALA file had no structural missingness for this field.
Clinical-type flags and deficiency tags	Percentages are based on the non-missing clinical/tag fields in each violation record. Records lacking a specific flag are treated as "flag = 0" (i.e., not that type).	The flag columns are binary one-hot indicators, so absence ≠ missingness.
HCUP Emergency-Department utilization	HCUP occasionally suppresses or suppresses certain payer strata when visit counts < 11 (cell-size rule). Those suppressed cells are coded "." in the file. For each state-year we: * summed whatever non-suppressed cell counts were available to get "all payers"; * calculated payer shares using only the available numerators & the *same* denominator. No statistical imputation was added.	Cell suppression is rare, affects only < 1 % of state-year-cells, and using available counts keeps analyses conservative (shares are slightly downward-biased toward 0).
State means across years (2018 → cut-off)	If a particular state lacks ED data for one of the years (e.g., 2020 COVID reporting gaps), the state-level mean is the average of the *available* years only. A state is still retained so long as ≥1 year of data exists in the window.	Averaging over available years preserves the panel structure without manufacturing values.

No forward/backward fills, mean substitutions, multiple imputation, or synthetic weighting were applied.

eMethods 1: Emergency Department (ED) Data Completeness and Missingness

Emergency department utilization data were derived from the Healthcare Cost and Utilization Project (HCUP) Fast Stats tool and reflect state-level ED visits by payer category. These include visits billed to Medicaid, self-pay/no-charge, and all payers combined. Data were reported quarterly. Data were incomplete for several states. Missingness varied by:

- Payer category (e.g., some states reported Medicaid but not self-pay volumes),
- Year or quarter (e.g., 2023 data were often incomplete or lagged),
- State participation in HCUP (not all states contribute all data streams).

Observations with missing ED volume for a given quarter and payer category were excluded listwise; no imputation or data filling was applied. Because HCUP data were not available through the whole post-policy study period, post-Dobbs follow-up was limited and ED analyses were considered secondary. Stratified models (e.g., by Medicaid expansion status) used only quarters and states with valid data to preserve the reliability of difference-in-differences estimates and avoid bias from interpolation. For all payer-related calculations, group summaries reflected only complete, observed data

© 2024 Woskie LR et al. JAMA Health Forum.

Medicaid Expansion

eMethods 2. Rationale for Including State Medicaid Expansion Status in Models of ED Utilization Related to Obstetric-Related EMTALA Violations

Disaggregation by Medicaid expansion status was used because the Affordable Care Act (ACA) expansion may plausibly modify the emergency-care environment in ways that can influence the likelihood of Emergency Medical Treatment and Labor Act (EMTALA) citations related to obstetric care, even though pregnancy-specific Medicaid eligibility itself is unaffected by expansion. Three inter-related mechanisms underlie this decision:

1. Emergency-Department (ED) Case-Mix, Volume, and Crowding
States that adopted the ACA expansion experienced markedly smaller increases in uninsured or self-pay ED visits and saw a shift toward insured payer mixes after 2014. Changes have been associated with reduced crowding and shorter boarding times, operational pressures linked to screening- and stabilization-related EMTALA infractions. In our own stratified analyses, post-*Dobbs* spikes in ED volume were appreciably larger in non-expansion states, underscoring the potential for confounding if expansion status is ignored.

2. Insurance Coverage Before, Between, and After Pregnancies
Medicaid expansion closes the "coverage gap" for low-income adults who are not yet recognized as pregnant or who have passed the traditional 60-day postpartum eligibility window. Earlier and more continuous insurance attachment may lessen delayed presentation and reduce reliance on the ED for emergent obstetric care, scenarios that can precipitate EMTALA breaches.

3. Hospital Financial Margins and Compliance Resources
By lowering uncompensated-care burdens, expansion can strengthen hospital operating margins. Health systems in expansion states may be better able to (a) maintain 24/7 obstetrics–gynecology call coverage, (b) allocate time for EMTALA-specific training, and (c) retain dedicated compliance personnel, each a plausible determinant of citation risk.

Consistent with this conceptual pathway, a binary indicator for state Medicaid expansion is included only as a covariate (and in stratified sensitivity checks) to address the possibility that broader post-ACA shifts in emergency-care delivery confound the estimated association between post-*Dobbs* abortion bans and obstetric-related EMTALA violations. The variable does not define exposure or treatment status and is not central to the study hypothesis; rather, it helps ensure that observed policy effects are not driven by concurrent differences in ED volume, patient mix, or hospital compliance capacity attributable to Medicaid expansion.

eTable 5. Medicaid Expansion Status by State

State	ACA Medicaid expansion in effect?	Implementation date
Idaho	Yes	Jan 1 2020
Kentucky	Yes	Jan 1 2014
Louisiana	Yes	Jul 1 2016
Oklahoma	Yes	Jul 1 2021
Mississippi	No	—
Texas	No	—

© 2024 Woskie LR et al. JAMA Health Forum.

eTable 6. Potential Mechanisms Underlying post-Policy ED Trends in Texas and Mississippi

Mechanism	Potential Relevance for Mississippi & Texas ED Increase in 2022
Post-Dobbs spill-over of pregnancy-related care	Stand-alone reproductive-health clinics that provided routine contraception, early-pregnancy, and gynecologic services closed or curtailed operations. Patients who would previously have been managed in those outpatient settings, especially uninsured or under-insured women, were more likely to appear in EDs for pregnancy confirmation, bleeding, miscarriage management, or requests for out-of-state referral.
Pandemic rebound & Omicron wave	ED volumes were still depressed in 2020-21. Omicron (winter 2021-22) produced a large, short-lived spike in ED use, and thereafter many deferred medical and surgical cases returned. Because Texas has a very large population base, even a modest per-capita rebound translates into tens of thousands of additional encounters.
High baseline uninsurance	Without Medicaid expansion, Mississippi and Texas maintained the highest uninsured rates in the country. The ED therefore may remain the default point of entry for primary and urgent care; any systemic shock, clinic closures, respiratory-virus surges, housing displacement, may show up disproportionately in ED counts.
Net in-migration and population growth (Texas)	Texas gained >400,000 residents between 2020 and 2022; raw visit counts naturally rise with population even if per-capita use is flat.
Payer-mix re-classification	The table shows a simultaneous rise in Medicaid & self-pay visits, consistent with encounters being re-coded once pregnancy-related eligibility was confirmed or postpartum coverage extensions were piloted. That coding shift may inflate the all-payer total even if the underlying clinical demand grew more modestly.

© 2024 Woskie LR et al. JAMA Health Forum.

Characterizing State Policy

eMethods 3. Identification of State Abortion Policies that Conflict with EMTALA

1.1 Conceptual definition: We defined a "No Meaningful Health Exception" statute as a near-total abortion prohibition that:

1. Is effectively comprehensive across gestation (eg, early cardiac-activity or total prohibitions) (trigger ban, pre-Roe revival, or SB-8 analogue);
2. Allows care only to prevent maternal death *or* avert a "substantial and irreversible impairment of a major bodily function"; mental-health indications are excluded or omitted;
3. Triggered a contemporaneous and documented allegation of conflict between state law and federal EMTALA guidance (eg, CMS deficiency letter, DOJ complaint, state Attorney-General advisory, or institutional policy bulletin citing EMTALA); and
4. Remained operative for ≥ 3 consecutive quarters during 2018 Q1–2023 Q1 (to ensure sufficient post-intervention follow-up).

1.2 Legal search strategy

Databases. Westlaw Edge Statutes & Regulations library (state & session laws); PACER, Bloomberg Law, and DOJ press releases (litigation); CMS online survey & certification database (deficiency letters).

	**Keyword String**
**Statutes**	(abortion) AND (ban OR prohibit OR trigger OR felony) AND ("medical emergency" OR "serious health risk" OR "irreversible impairment" OR "major bodily function" OR "life-sustaining organ") & date = 2018-01-01 to 2023-03-31
**EMTALA Conflict**	(EMTALA OR "Emergency Medical Treatment and Labor Act") AND (abortion OR obstetr*) AND (Idaho OR Kentucky OR …) & date = 2018-01-01 to 2023-03-31

Study flow:

1. Capture universe of near-total bans (Step 1).
2. Extract operative exception text; highlight phrases narrower than EMTALA's "serious jeopardy" standard (Step 2).
3. Link each statute to conflict evidence (CMS letter, federal complaint, AG guidance) (Step 3).
4. Apply stability screen—exclude bans never enforced for ≥ 3 continuous quarters (Step 4).
5. Populate timeline spreadsheet with effective dates, injunctions, amendments (Step 5).

© 2024 Woskie LR et al. JAMA Health Forum.

eMethods 4. Overview of Sources & Processes to Characterize State Policy

State policy abstraction proceeded in four sequential phases:

1) First, we constructed a comprehensive universe of near-total abortion prohibitions enacted or operative between January 1 2018 and March 31 2023. Using Westlaw Edge's *Statutes & Regulations* library, we queried the full text of every state code for the terms *abortion* AND (*ban* OR *prohibit* OR *trigger* OR *felony*) AND key exception phrases (eg, *medical emergency*, *serious health risk*, *irreversible impairment*, *major bodily function*, *life-sustaining organ*). We downloaded the enrolled-bill or slip-law version of each hit from the corresponding state-legislative website and double-checked operative language and effective-date clauses against three independent policy trackers, the Guttmacher Institute's "State Bans on Abortion," the Kaiser Family Foundation State Policy Tracker, and the Center for Reproductive Rights "After Roe" map, to ensure textual and temporal accuracy.

2) Second, we linked each statute to publicly documented evidence that the law had come into conflict with the Emergency Medical Treatment and Labor Act (EMTALA). To do so, we searched (1) the Centers for Medicare & Medicaid Services (CMS) Survey & Certification portal for deficiency letters citing EMTALA and abortion; (2) PACER and Bloomberg Law dockets, as well as US Department of Justice press releases, for federal pre-emption actions; (3) state attorney-general press rooms for advisories or formal opinions referencing EMTALA; and (4) LexisNexis and hospital-association newsletters for institutional compliance bulletins. A statute advanced to the treatment candidacy list only if at least one EMTALA-related document was identified.

3) Third, we applied a stability screen: statutes that were entirely enjoined, vacated, or otherwise inoperative for three or more consecutive quarters were removed from the treatment set to guarantee sufficient post-implementation observation time for difference-in-differences estimation. This criterion was operationalized by tracing injunction and amendment timelines through Bloomberg Law's "Track Legislation" service and cross-validating dates with the aforementioned policy trackers.

4) Finally, we abstracted and coded statutory text, conflict documentation, and enforcement timelines into a master spreadsheet. Discrepancies were reviewed by an external reviewer with formal legal training. The resulting classification yielded six treated states (Idaho, Kentucky, Louisiana, Mississippi, Oklahoma, Texas), seven "potentially treated" but ultimately excluded states, and a control cohort comprising the remaining jurisdictions with qualifying hospital-year observations.

© 2024 Woskie LR et al. JAMA Health Forum.

eTable 7. Application of Inclusion / Exclusion Rules, 2018 Q1–2023 Q1

Group	State(s)	Statutory language in force	EMTALA conflict evidence	Meets ≥ 3-quarter stability?
**Treatment (N = 6)**<br><br>NMHE-EC bans	**Idaho**	Idaho Code §18-622: abortion felony unless to prevent death. Defines "medical emergency" as death risk or *irreversible impairment of major bodily function.*	*United States v Idaho* (DOJ pre-emption suit 2022); CMS July 2022 memo to Idaho hospitals.	Yes (2022 Q3–2023 Q1)
	**Kentucky**	Ky. Rev. Stat. §311.772: exception only for death or *substantial and irreversible impairment.*	AG Cameron letter to CMS (Aug 2022) disputing EMTALA guidance.	Yes (2022 Q3–2023 Q1)
	**Louisiana**	La. Rev. Stat. §40:1061.1.5: permits abortion to avoid death or *serious, permanent impairment of a life-sustaining organ.*	Ochsner Health EMTALA compliance bulletin (Sept 2022); AG Landry advisory (July 2022).	Yes (2022 Q3–2023 Q1)
	**Mississippi**	Miss. Code Ann. §41-41-45: death-only exception.	CMS deficiency findings ref. EMTALA, Univ. of Miss. Med. Ctr. (Oct 2022).	Yes (2022 Q3–2023 Q1)
	**Oklahoma**	63 Okla. Stat. §1-731.4 & HB 4327: abortion barred except to save life in "medical emergency."	OU Health system EMTALA alert (June 2022).	Yes (2022 Q2–2023 Q1)
	**Texas**	Tex. Health & Safety Code §§171.205, 170 A.002: exception for death or *substantial impairment of a major bodily function.*	HHS/CMS EMTALA guidance letter (July 2022) naming Texas; *Moyle v U.S.* consolidated appeal.	Yes (2021 Q3–2023 Q1)
**Potentially Treated (N = 7)**	**Arkansas, South Dakota**	Life-only trigger bans; **no EMTALA citation** in 2018-2023 and < 5 obstetric investigations → fail Criterion 3 & 4.	—	No
	**Alabama, North Dakota**	"Serious health risk" bans omit "irreversible/permanent" language; no EMTALA action → fail Criterion 2 & 3.	—	n/a
	**Missouri, Tennessee, West Virginia**	Use "irreversible" phrasing but first EMTALA-based suits **filed in 2024**, outside window → fail Criterion 3.	n/a	n/a

© 2024 Woskie LR et al. JAMA Health Forum.

**Policy-volatile controls (N = 6)[a]**	**Arizona, Florida, Georgia, Indiana, Utah, Wyoming**	Multiple injunctions & reinstatements (≥3 legal flips) → violate stability rule.	—	No
**Stable controls (N = 29 + DC)**	**All remaining jurisdictions**	No near-total ban; gestational limits retain broad physical **and** mental-health exceptions aligned with EMTALA.	—	n/a

[a]Several states with early cardiac-activity limits (eg, Georgia) or other partial restrictions were not coded as treated because they retained explicit physical-health exceptions, OR lacked documented allegations of conflict between state law and the federal EMTALA mandate, OR remained operative for fewer than three consecutive quarters during the study period. These jurisdictions were combined with stable controls to preserve a consistent comparison group and avoid over-weighting small or policy-volatile states. However, such laws merit further examination; they may influence emergency care similarly to those in the treated group. Their exclusion likely biases estimates toward the null.

© 2024 Woskie LR et al. JAMA Health Forum.

eTable 8. "Documented EMTALA conflict" as inclusion criteria

	Rationale
Documented conflict allegation	A contemporaneous, public record showing that state officials, hospitals, or the U.S. Department of Health & Human Services (HHS) explicitly acknowledged that a state's abortion ban could prevent clinicians from meeting EMTALA's *stabilize-the-patient* mandate.<br><br>Typical sources are DOJ pre-emption complaints or attorney-general guidance memoranda.
Why is this evidence legally salient?	Under the Supremacy Clause, federal law pre-empts conflicting state law. EMTALA is a federal statute requiring emergency departments to provide stabilizing treatment when the patient's health is "in serious jeopardy." If state abortion law bars that treatment, a *conflict pre-emption* question arises. Documentation of alleged conflicts events therefore establish that the pre-emption issue is not hypothetical but formally recognized by at least one competent authority.
Relevance to study design?	1. Exposure specificity. It limits the treatment group to bans that plausibly *interfere* with EMTALA-mandated care, rather than all bans with narrow wording.<br>2. Mechanism alignment. The outcome (obstetric-related EMTALA violations) and the exposure (a statute already flagged as conflicting with EMTALA) lie on the same legal-clinical pathway, sharpening causal inference.<br>3. Avoids dilution. States whose bans never triggered EMTALA scrutiny may either have de facto broader practice patterns or insufficient enforcement.
Testing pre-emption in practice?	By observing whether EMTALA violations *increase* after a conflict-flagged ban takes effect, the analysis assesses whether federal pre-emption is functioning on the ground. A significant rise suggests that, despite the Supremacy Clause, the state law is constraining clinical behavior and EMTALA enforcement does not fully offset the conflict.<br><br>Without evidence that EMTALA was *invoked* against the state law, we cannot distinguish:<br>a.) Narrow statutory text that hospitals ignore (no real conflict) and<br>b.) Narrow text that alters care (true conflict).<br>The documented-conflict filter allows us to actively study the latter.

© 2024 Woskie LR et al. JAMA Health Forum.

Pre-intervention Lead Coefficients & Joint Parallel-Trend Tests

eTable 9. Joint Parallel-Trend Test for the Primary Obstetric-Related Difference-in-Differences Model

Relative quarter before treatment	β (SE)	P value
–4 quarters	*–0.07 (0.15)*	*.63*
–3 quarters	*+0.05 (0.13)*	*.70*
–2 quarters	*–0.06 (0.12)*	*.61*

Estimates shown are the coefficients for the three pre-treatment leads; the Wald test evaluates the joint null that all lead coefficients equal 0.
Abbreviations: CI, confidence interval; EMTALA, Emergency Medical Treatment and Labor Act; SE, standard error.

eTable 10. Tag-Specific "Violation Type" Difference-in-Differences Models

Tag (code)	β-4 q (SE), *p*	β-3 q (SE), *p*	β-2 q (SE), *p*	Wald test, all pre-period = 0
Medical-screening exam (2406)	–0.04 (0.07), .59	–0.02 (0.06), .71	+0.03 (0.05), .53	χ^2 = 1.2, df = 3, *p* = .76
General compliance (2400)	–0.01 (0.06), .85	+0.06 (0.05), .25	+0.02 (0.06), .73	χ^2 = 1.8, df = 3, *p* = .62
Appropriate transfer (2409)	+0.01 (0.03), .70	–0.01 (0.03), .77	–0.02 (0.04), .57	χ^2 = 0.9, df = 3, *p* = .82
Stabilizing treatment (2407)	–0.05 (0.10), .62	+0.03 (0.09), .77	–0.04 (0.09), .66	χ^2 = 0.6, df = 3, *p* = .89
ED-log maintenance (2405)	+0.02 (0.05), .67	–0.03 (0.04), .48	+0.01 (0.05), .86	χ^2 = 1.1, df = 3, *p* = .78

Each model replicates the primary Model III specification—state-quarter fixed effects and emergency-department volume covariate—using the quarterly count of the indicated EMTALA deficiency tag within obstetric-related violations as the outcome. Estimates show the difference between treated and control states for the 4th, 3rd, and 2nd quarters before each state's abortion-ban start. The Wald test evaluates the joint null that all pre-period coefficients equal 0. Standard errors are clustered by state.
Abbreviations: χ^2, chi-square statistic; EMTALA, Emergency Medical Treatment and Labor Act; SE, standard error.

© 2024 Woskie LR et al. JAMA Health Forum.

eTable 11. Point estimates underlying Figure 1, primary DiD models

Outcome	Model	Specification†	Δ violations per state-quarter (95 % CI)	*p*-value
**Obstetric**	1	None (unadjusted)	**+ 0.50** (0.09 to 0.90)	.016
	2	+ State FE + Quarter FE	**+ 0.70** (0.19 to 1.20)	.007
	3	Model 2 + ED-visit volume	**+ 1.18** (0.49 to 1.86)	.001
**Medical**	1	None	– 0.10 (– 1.58 to 1.39)	.900
	2	+ State FE + Quarter FE	+ 0.12 (– 1.43 to 1.67)	.882
	3	Model 2 + ED-visit volume	+ 0.35 (– 2.00 to 2.69)	.771
**Surgical**	1	None	+ 0.08 (– 0.04 to 0.20)	.209
	2	+ State FE + Quarter FE	+ 0.18 (– 0.02 to 0.37)	.071
	3	Model 2 + ED-visit volume	+ 0.21 (– 0.11 to 0.53)	.201
**Psychiatric**	1	None	+ 0.29 (– 0.96 to 1.54)	.650
	2	+ State FE + Quarter FE	– 0.61 (– 1.69 to 0.47)	.266
	3	Model 2 + ED-visit volume	– 1.00 (– 3.16 to 1.17)	.369

© 2024 Woskie LR et al. JAMA Health Forum.
