# Supplementary material for: Obstetric-Related Emergency Medical Treatment and Labor Act Violations and No Health Exception Bans
Source: JAMA Health Forum. 2025 Dec 5;6(12):e254726. doi: 10.1001/jamahealthforum.2025.4726 (PMC12681040; doi:10.1001/jamahealthforum.2025.4726)
Supplement: Supplement 2. — Data sharing statement [file jamahealthforum-e254726-s002.pdf]

Data Sharing Statement

Woskie. Obstetric-Related Emergency Medical Treatment and Labor Act Violations and No Health Exception Bans. *JAMA Health Forum*. Published December 05, 2025. doi:10.1001/jamahealthforum.2025.4726

Data
Data available: No

Additional Information
Explanation for why data not available: The data used in this study were obtained via a Freedom of Information Act (FOIA) request from the Centers for Medicare & Medicaid Services (CMS). At present, we are not authorized to publicly share the dataset but intend to make a cleaned version publicly available in a repository upon final review and clearance. We are committed to transparency and will update data availability accordingly.
